# Substantial deletion overlap among divergent *Arabidopsis *genomes revealed by intersection of short reads and tiling arrays

**DOI:** 10.1186/gb-2010-11-1-r4

**Published:** 2010-01-12

**Authors:** Luca Santuari, Sylvain Pradervand, Amelia-Maria Amiguet-Vercher, Jerôme Thomas, Eavan Dorcey, Keith Harshman, Ioannis Xenarios, Thomas E Juenger, Christian S Hardtke

**Affiliations:** 1 Department of Plant Molecular Biology, University of Lausanne, Biophore Building, CH-1015 Lausanne, Switzerland; 2 Swiss Institute of Bioinformatics, Genopode Building, CH-1015 Lausanne, Switzerland; 3 Lausanne DNA Array Facility, Center for Integrative Genomics, University of Lausanne, Genopode Building, CH-1015 Lausanne, Switzerland; 4 Section of Integrative Biology and Institute for Cellular and Molecular Biology, The University of Texas at Austin, 1 University Station C0930, Austin, Texas 78712, USA

## Abstract

Identification of small polymorphisms from next generation sequencing short read data is relatively easy, but detection of larger deletions is less straightforward. Here, we analyzed four divergent *Arabidopsis *accessions and found that intersection of absent short read coverage with weak tiling array hybridization signal reliably flags deletions. Interestingly, individual deletions were frequently observed in two or more of the accessions examined, suggesting that variation in gene content partly reflects a common history of deletion events.

## Abstract 2

A new approach to detect deletions in divergentgenomes combines short read sequencing and tilling array data. Its utility is demonstrated on *Arabidopsis *strains.

## Background

Ultra-high throughput sequencing (UHTS) has become affordable to re-sequence genomes of model organisms, such as *Arabidopsis thaliana *[1-5]. While identification of single nucleotide polymorphisms (SNPs) and small indels from UHTS short reads is relatively easy, detection of structural variation, such as larger deletions, is less straightforward [2,3,6,7]. This is particularly true for analysis of divergent genomes, such as those of *Arabidopsis *strains that are not closely related to the reference accession, Columbia-0 (Col-0). For instance, the accuracy of short read mapping depends on the number of polymorphic sites permitted per read [8]. If it is set too high, it can result in read mapping to false locations; if it is set too low, it can prevent mapping to the correct location. Moreover, local accumulation of polymorphisms with respect to the reference genome can occur and such reads could only be correctly mapped with unrealistically relaxed settings that would interfere with overall correct annotation. Consequently, the corresponding reference genome regions would not be covered in standard mapping protocols, and whether or not these regions reflect excess polymorphism or deletions would remain ambiguous. Novel technologies, such as paired end read sequencing, combined with novel instruments, might eventually enable precise mapping of larger deletions. However, to date bioinformatic tools to exploit such data are still scarce [6], and whether the available algorithms deliver comprehensive analyses has not been experimentally verified.

Another tool to predict deletions are genome tiling array hybridizations, either through statistical analysis of hybridization signals [9-11] or empirically determined thresholds [12,13]. In these approaches, signal ratios from hybridizations with DNA from a divergent strain versus DNA from the reference strain used for array design are analyzed to infer absence of the sequence homologous to a given tile. However, experimental verification suggests that deletions predicted in this manner contain a high number of false positives (approximately 47%) [13].

Finally, although inherently difficult and non-comprehensive [14,15], contig-building from UHTS could identify larger deletions in genome variants with some success [3]. Interestingly, these correlated with reduced hybridization signal in corresponding re-sequencing arrays [3,7,16]. Thus, intersection of UHTS with tiling array hybridization could be a powerful tool to pinpoint deletions. Here we applied this procedure to investigate genomic variation in four divergent, isogenized *Arabidopsis *strains (so-called accessions): Eilenburg-0 (Eil-0), Loch Ness-0 (Lc-0), Slavice-0 (Sav-0) and Tsushima-1 (Tsu-1).

## Results and discussion

Novel UHTS data were generated for Eil-0, Lc-0 and Sav-0 using an Illumina Genome Analyzer II platform, while published data for Tsu-1 [3] served as comparison. To estimate the quality of our data, we mapped the Eil-0 and Lc-0 short reads onto previously established approximately 94 kb (Eil-0) and approximately 96 kb (Lc-0) of high quality genomic DNA sequence obtained from 144 loci by dideoxy sequencing [12]. Mapping with three mismatches allowed in the 5', 28 bp of each 35- to 36-bp read to account for sequencing errors using MAQ (Mapping and Assembly Quality software) [17] failed to cover approximately 1.3% (Eil-0) and 5.0% (Lc-0) of sequence, which thus appeared to be absent. Such missing sequence is not unusual and could reflect insufficient coverage (17.1 for Eil-0, 6.4 for Lc-0), the stochastic nature of the sequencing process, or technical biases [3,5,18-20].

**Figure 1:**
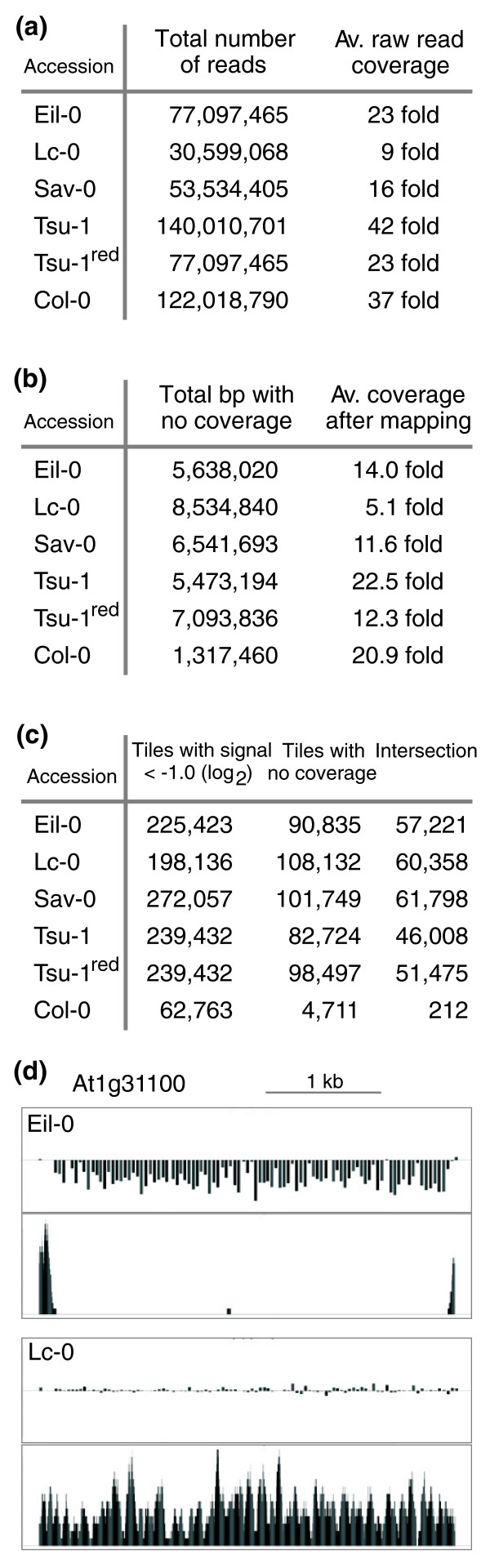
**UHTS and tiling array statistics for the investigated accessions**. **(a) **Total number of short reads (35 bp for paired end runs; 36 bp for single end runs) obtained for each accession after quality filtering and calculated raw coverage (single end runs were performed for Eil-0, Lc-0 and Sav-0; additional paired end runs for Eil-0 and Lc-0; Tsu-1 and Col-0 reads from single end runs were obtained from published data). For Tsu-1, a subset of reads was retrieved (Tsu-1^red^) for comparative purposes. **(b) **Average coverage after MAQ mapping of the short reads onto the Col-0 reference genome and number of base-pairs in the reference genome with zero coverage. **(c) **Genomic tiling array statistics. Left: number of unique tiles with relative hybridization signal ratio <-1.0 (log_2_) calculated from the averages of two array hybridizations with divergent DNA versus two array hybridizations with the reference DNA. Middle: number of unique tiles with no UHTS coverage across all 25 bp of the tile. Right: intersection between the two groups of tiles. **(d) **Example plot of tiling array signal ratio (top panel) versus UHTS coverage (bottom panel). The entire gene (At1g31100) appears to be deleted in Eil-0, but appears to be intact in Lc-0. Please refer to Figure 3c for detailed plot labels.

Mapped onto the Col-0 reference sequence [21], the Eil-0, Lc-0, Sav-0 and Tsu-1 UHTS reads failed to cover approximately 5.6 Mb, 8.5 Mb, 6.5 Mb and 5.5 Mb, respectively (Figure 1a). Average coverage after mapping was approximately 14.0 (Eil-0), 5.1 (Lc-0), 11.6 (Sav-0) and 22.5 (Tsu-1) (Figure 1b). Similar mapping of Col-0 short reads obtained from re-sequencing [3] also could not cover approximately 1.3 Mb (average coverage approximately 20.9), suggesting that in the divergent accessions, portions of the genome escaped UHTS or were too polymorphic to be correctly mapped. Insufficient coverage could be one reason as mapping a subset of the Tsu-1 reads, equaling the number of Eil-0 reads, increased the non-covered sequence from approximately 5.5 Mb to 7.1 Mb (Figure 1b). However, insufficient coverage could not explain all missing homologous sequence, as estimated by the lower end of coverage distribution (Additional file 1). Notably, this distribution did not follow gamma or Poisson distributions that were recently used to model coverage of short read sequences [3,22]. Thus, portions of the reference sequence must indeed be missing in the accessions. Which exactly is difficult to determine, however, because of bioinformatic constraints on short read mapping [3,14,15,18,23]. To overcome these limitations, we sought to complement UHTS by an independent approach and thus intersected our short read mappings with tiling array hybridizations.

Using available tiling array data [12] and additional hybridizations, we determined the hybridization signal ratio (that is, log_2 _of mean signal from two arrays hybridized with divergent DNA divided by mean signal from two arrays hybridized with Col-0 DNA) of all 25-bp tiles (Affymetrix *Arabidopsis *Tiling 1.0R Arrays) for each accession. To avoid ambiguities due to cross-hybridization, we concentrated on tiles that are unique in the Col-0 genome [9]. Next, we determined the tiles' UHTS coverage based on our MAQ mappings. Tiles that were not at all covered were considered candidates for missing sequence and analyzed further. We first applied an empirically determined threshold [12] and selected tiles with a signal ratio less than -1.5. To detect major deletions, we focused on consecutive tiles that covered ≥300 bp (taking into account spacing between tiles, typically 10 bp). For experimental verification, we chose 47 deletions predicted on chromosome 1 of Eil-0 (26) or Tsu-1 (21) and designed flanking primers (Additional file 2). In replicate PCR experiments with independent genomic DNA template preparations, we then observed a consistent pattern: nearly all (46) loci could be amplified from Col-0 DNA as expected; by contrast, loci presumptively deleted in Eil-0 could not be amplified from Eil-0 DNA, but could be amplified from Tsu-1 DNA, and *vice versa*; loci presumptively missing from both Eil-0 and Tsu-1 could not be amplified from either background. Inspection of the tiles flanking the loci, up to and beyond primer locations, revealed that they were often not covered and had negative, although not <-1.5, signal ratios (average -0.85 for Eil-0, -1.22 for Tsu-1). Thus, our criteria were apparently overly stringent. The particular threshold used should be driven by the goals of particular researchers and the cost associated with false positive or false negative inferences (Additional file 3). In the following, we focused on an empirical threshold of less than -1.0 derived from the signal ratios describe above and the average ratios from polymorphic tiles in the Eil-0 and Lc-0 dideoxy reference sequences. This simple threshold identified a set of putative deletions with high confidence.

To estimate technical variability, we first intersected Col-0 tiling array hybridizations and UHTS data [3]. Out of 2.88 million tiles considered, 62,720 displayed a signal ratio <-1.0, and 4,711 could not be covered by UHTS reads (Figure 1c). The intersection of the two groups was only 212 tiles. Considering the range of intersection in the four accessions (46,008 to 61,798 tiles), false positives due to technical variability thus appeared to be relatively low. In the divergent genomes, a significant fraction of intersection tiles might represent SNP hotspots that could not be mapped [7]. Interestingly, such hotspots have been preferentially found around confirmed deletions in rice strains [24]. However, the fraction of such tiles should be relatively low, as even high levels of polymorphism (5 to 10 SNPs in 25 bp) resulted in rather mild negative signal ratio as determined from the dideoxy data (average -0.21). Moreover, based on the <-1.0 threshold, we selected 21 predicted deletions ≥100 bp from Lc-0, the accession with lowest UHTS coverage. For PCR verification, primers were this time designed to anneal in well covered flanking regions (Additional file 2). All 21 loci could be amplified and 17 displayed deletions in Lc-0. Thus, our method performed well even with limited UHTS data.

Approximately 57% of Eil-0 reads and all Lc-0 reads originated from paired end sequencing runs, which would principally enable direct prediction of deletions from paired end map positions. To estimate the performance of our approach, we thus re-analyzed the Eil-0 and Lc-0 reads using the Breakdancer algorithm [6], an extension of MAQ that takes into account spacing between mapped paired end reads to predict deletions. Interestingly, this approach generally predicted fewer deletions (Additional file 4) and failed to identify 2 out of 17 experimentally confirmed deletions in Lc-0, and 17 out of 26 in Eil-0 (Additional file 2). Importantly, this was true for repeated analyses that explored the Breakdancer parameter range. Thus, with our data, intersection of UHTS with tiling arrays yielded more comprehensive information, particularly with respect to larger deletions, such as those experimentally verified for Eil-0.

**Figure 2:**
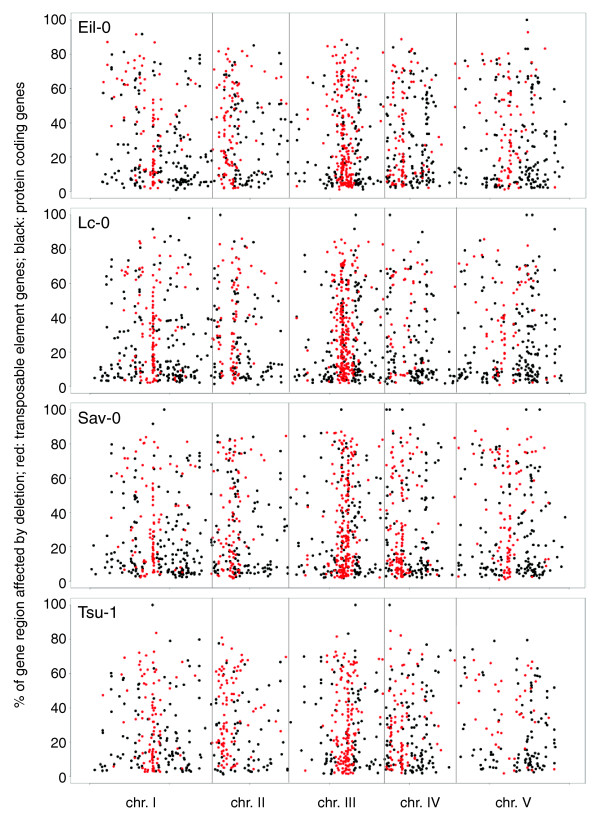
**Genome-wide distribution and size of deletions within genes**. Deletions were called by intersecting a tiling array signal ratio <-1.0 (log_2_) of individual 25-bp tiles with no coverage of the entire tiles by UHTS short reads, for at least 100 bp. Tiles within a gene that fulfilled these criteria were added up, taking into account the gaps between tiles (typically 10 bp; maximum 39 bp), to calculate the approximate proportion of a gene affected by a deletion(s) (y axis). Each dot represents a gene: red dots represent transposable element genes (which cluster around the centromeres); black dots represent protein coding genes. The genes are plotted along the five *Arabidopsis *chromosomes (chr.), drawn to scale (x axis).

Next, we mapped substantial putative deletions within genes - that is, no read coverage combined with a signal ratio <-1.0 for at least 100 bp. By these criteria, 1,220 (Eil-0), 1,312 (Lc-0), 1,344 (Sav-0) and 987 (Tsu-1) genes with deletions were identified (Additional file 5). Many of these deletions (36.6 to 41.4%) affect the coding region and thus likely impair gene activity (Additional file 6). As evident from plots of coverage versus signal ratio, tiles fulfilling our criteria frequently clustered and spanned significant portions of the genes (for example, Figure 1d). Moreover, they were often surrounded by tiles with no coverage and negative, although not <-1.0, signal ratios. Even with the <-1.0 threshold strictly maintained, many genes appeared to be affected by rather large deletions (Figure 2), which would eliminate significant portions of coding sequence.

**Figure 3:**
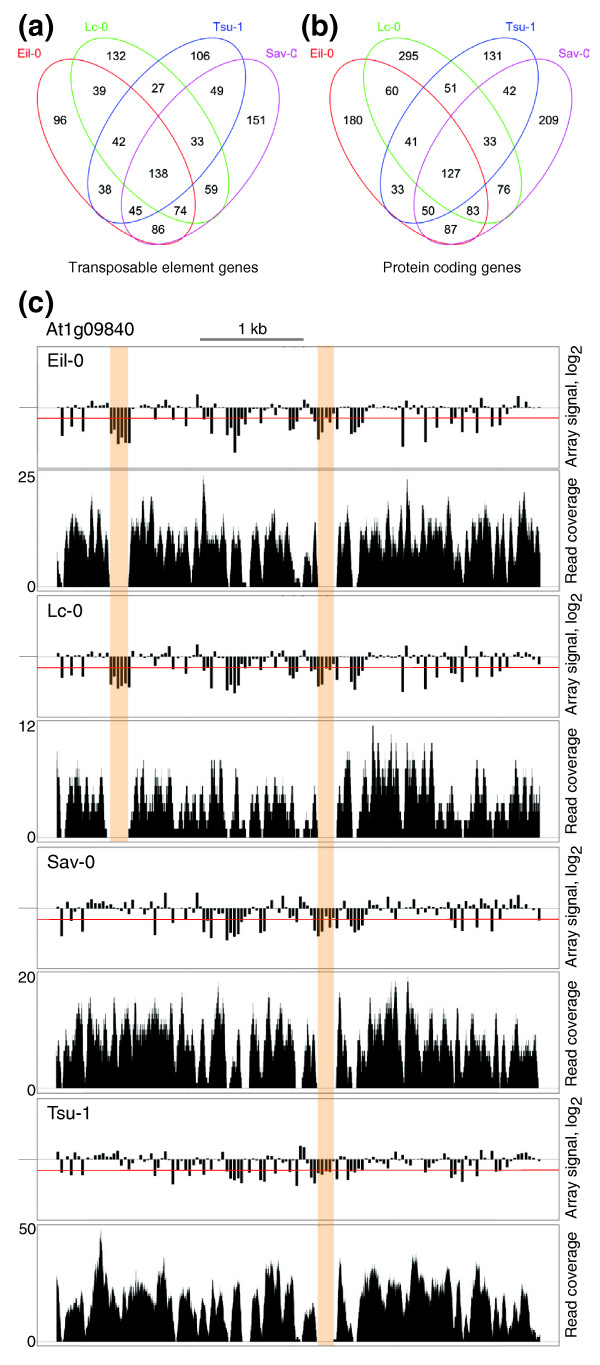
**Overlap of deletions between two or more of the four accessions examined**. **(a) **Venn diagram of the overlap between transposable element genes for which deletions (that is, tiling array signal ratio <-1.0 (log_2_) and no short read coverage for at least 100 bp) could be detected in the different accessions. **(b) **Same as (a), for protein coding genes. **(c) **Example plot of tiling array signal ratio versus UHTS short read coverage for a gene (At1g09840) in all four accessions. Top panels: tiling array signal ratio (log_2_), with the -1.0 threshold indicated by a red line. Bottom panel: corresponding short read coverage after MAQ mapping. A major deletion shared by two accessions (Eil-0 and Lc-0) and another shared by all four accessions are highlighted.

We also observed a strong bias in the distribution of deletions. Generally, genes annotated as transposable element genes were more abundant than expected (41.6 to 48.5% of all loci; that is, 3.3 to 3.9-fold over-represented; *P *< 0.001 [χ^2 ^statistic]), matching reports from SNP analyses [3,10,11]. Conversely, genes annotated as protein coding were under-represented. While bias towards transposable element genes could be expected given their role as generally non-essential genetic material, another observation was less obvious, namely large overlap between the genes with predicted deletions in the different genotypes. For the transposable element genes, only 17.2 to 24.3% of deletions were unique for a given accession, while all others were shared with at least one of the three other backgrounds (Figure 3a). More than one-third (38.6 to 45.2%) of genes were affected in at least three accessions, and 21.7 to 28.9% (n = 138) in all four genotypes. A similar pattern was evident for protein coding genes, although the proportion of uniquely affected genes was somewhat higher (25.8 to 38.5%) (Figure 3b). Still, a high amount (16.6 to 25.0%) of them was affected in all four backgrounds (n = 127). Although the exact extent of individual deletions would have to be determined by dideoxy sequencing, they frequently appeared to be roughly identical in the different accessions. Moreover, patterns of deletions were often shared between accessions (for example, Figure 3c), suggesting that they reflect a common ancestry and history of rearrangements.

## Conclusions

Our study suggests that combination of UHTS with tiling array analysis is a valid and economical approach to reliably flag deletions in divergent genomes. Analysis of the four divergent genomes suggests that deletions preferentially affect transposable element genes, but also significant numbers of protein coding genes. Our observation that many predicted deletions are shared between two or more of the accessions examined suggests that variation in gene content to some degree reflects a common history of deletion events, which has been partly shaped by transposable element activity.

## Materials and methods

### Tiling arrays: mapping and pre-processing

DNA samples (extracted with Qiagen [Hilden, Germany] DNeasy Plant kits according to the manufacturer's instructions) from the four accessions were hybridized to Affymetrix GeneChip^® ^*Arabidopsis *Tiling 1.0R arrays in duplicate as described [12]. Probe sequences from the BPMAP specification of the array (At35b_MR_v04-2_TIGRv5) were mapped on the Col-0 TAIR8.0 genome release downloaded from The *Arabidopsis *Information Resource (TAIR) [25], using BioConductor [26]. Only probes with a perfect match and single occurrence in the genome were retrieved. Approximately 15% of reads in each sample represented contamination from organelle DNA. For each accession, probe intensities from two tiling array hybridizations were normalized by quantile normalization along with the intensity values of the two reference array hybridizations of Col-0 DNA. The log_2 _ratio of the mean of the two intensities from the accession arrays over the mean of the two intensities from the control arrays was taken as the reference signal for each tile.

### UHTS: genome-wide mapping of short reads and coverage analysis

The genomes of Eil-0, Lc-0 and Sav-0 were re-sequenced using the Illumina Genome Analyzer II platform according to the manufacturer's instructions. Several lanes of either single end runs (Eil-0 and Sav-0) or paired end runs (Eil-0 and Lc-0) were produced for each accession. Short reads from single end runs for Col-0 and Tsu-1 were retrieved from published data [3]. For each accession, short reads were filtered by quality (MAQ standard settings) and mapped on the TAIR 8.0 Col-0 genome using the MAQ algorithm [17]. We allowed up to three mismatches in the first (5') 28 bp of the read. The number of reads mapped on each base-pair was considered in all subsequent analyses and we defined it as the read coverage. For each tile, we computed the mean coverage across the 25 bp interval on the genome relative to the probe sequence and we used this information in the comparison of the tiling array signal with the short read coverage. Purely bioinformatic deletion mapping taking into account the information from paired end data was performed using the Breakdancer algorithm [6], an extension to MAQ.

### Mapping of short reads to the Eil-0 and Lc-0 dideoxy reference sequence

Short reads from the Eil-0 accession were mapped onto 94,076 bp of dideoxy sequence obtained from 144 loci of the Eil-0 genome, onto 95,980 bp of dideoxy sequence obtained from 144 loci of the Lc-0 genome using the MAQ software. We allowed from zero up to three mismatches in the mapping process to take into account possible sequencing errors. We performed five repetitions in order to see how much the reads with several possible mapping positions on the reference sequence affect the coverage.

### Identification of deletions and gene level analysis

For each of the four accessions, we analyzed the mean coverage and the signal relative to the genomic positions of the probe sequence of each tile. We identified regions where the probe sequences are spaced by typically 10 bp, but always less than 40 bp, and are characterized by having no short read coverage and a tile signal ratio below an arbitrary threshold. According to the analysis of the distribution of the signal in each array, at first we decided to set this threshold to be <-1.5. After PCR validation of major deletions in Eil-0 and Tsu-1, we were able to determine a less stringent threshold, <-1.0, and we repeated the above analysis to annotate putative deletions for each strain. To understand how these deletions affect functional gene content in the accessions, we considered the base-pair positions of the deleted regions that span the genes, based on the TAIR 8.0 GFF gene annotation. We first analyzed untranslated regions, exons and introns, according to the 'mRNA' feature in the GFF annotation file, and then focused on the coding sequences of the genes, the 'CDS' (coding sequence) feature in the GFF file.

### Molecular biology and plant materials

Plant tissue culture and molecular biology procedures followed routine protocols as described [12,27]. Tiling array source files are available from ArrayExpress [ArrayExpress:E-MEXP-2220], all short reads generated in this study are available from the NCBI-GEO short read archive [NCBI-GEO:SRA009330]. The scripts used for the bioinformatics analyses of our data are provided in Additional files 7 and 8.

## Abbreviations

Col-0: *A. thaliana *accession Columbia-0; Eil-0: *A. thaliana *accession Eilenburg-0; Lc-0: *A. thaliana *accession Loch Ness-0; MAQ: Mapping and Assembly Quality software; Sav-0: *A. thaliana *accession Slavice-0; SNP: single nucleotide polymorphism; TAIR: The *Arabidopsis *Information Resource; Tsu-1: *A. thaliana *accession Tsushima-1; UHTS: ultra-high throughput sequencing.

## Authors' contributions

CSH, LS, KH, IX and TEJ conceived this study and analyzed the data with help from SP. CSH wrote the manuscript together with LS and TEJ; JT performed the UHTS sequencing runs; ED contributed the Sav-0 tiling array data; AMAV experimentally verified deletions; all bioinformatics analyses were performed by LS. All authors read and approved the final manuscript.

## Supplementary Material

Additional file 1 Distribution of the read coverage for the five *Arabidopsis *chromosomes across the different accessions. Click here for file

Additional file 2 Experimentally tested deletions predicted in Eil-0 and Tsu-1, positions and primer sequences. Click here for file

Additional file 3 Number of unique tiles from the Col-0 genome without UHTS coverage in the four accessions for different tiling array signal ratios. Click here for file

Additional file 4 Deletions predicted from paired end reads of the Eil-0 and Lc-0 genomes by the Breakdancer algorithm. Click here for file

Additional file 5 Genes carrying deletions as predicted from intersection of UHTS and tiling arrays. Click here for file

Additional file 6 The subset of genes whose coding sequence is carrying deletions as predicted from intersection of UHTS and tiling arrays. Click here for file

Additional file 7 Script for computing the UHTS coverage and signal ratio of array tiles. Click here for file

Additional file 8 Script for deletion prediction based on intersection of UHTS coverage and tiling array signal. Click here for file
