# Supplementary material for: Substantial deletion overlap among divergent Arabidopsis genomes revealed by intersection of short reads and tiling arrays
Source: Genome Biol. 2010 Jan 12;11(1):R4. doi: 10.1186/gb-2010-11-1-r4 (PMC2847716; doi:10.1186/gb-2010-11-1-r4)
Supplement: Additional file 1 — Distribution of the read coverage for the five Arabidopsis chromosomes across the different accessions. [file gb-2010-11-1-r4-S1.pdf]

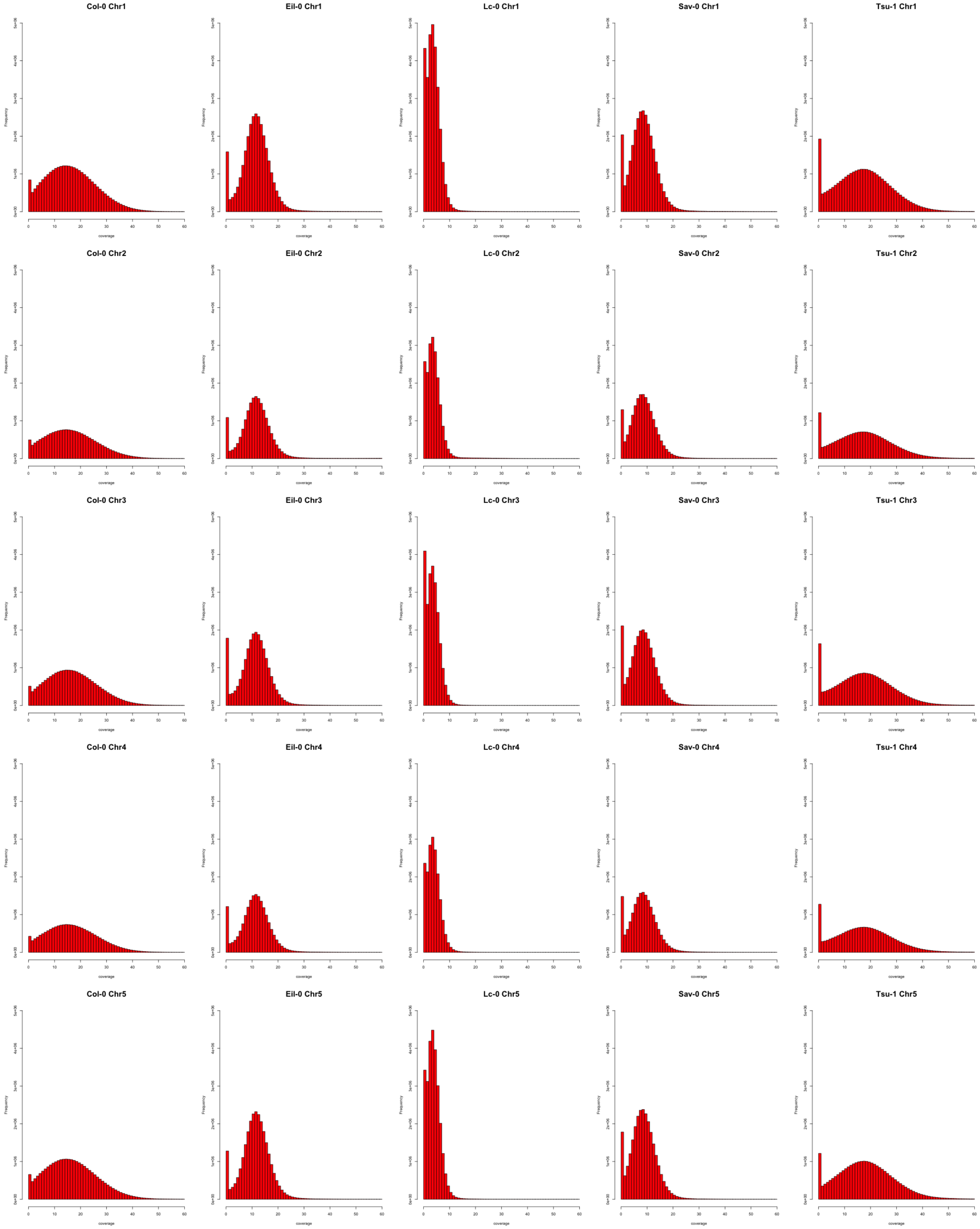

Supplemental Figure 1 Distribution of the read coverage for the five Arabidopsis chromosomes across the different accessions. y axis: frequency; x axis: coverage;
