# Supplementary material for: Substantial deletion overlap among divergent Arabidopsis genomes revealed by intersection of short reads and tiling arrays
Source: Genome Biol. 2010 Jan 12;11(1):R4. doi: 10.1186/gb-2010-11-1-r4 (PMC2847716; doi:10.1186/gb-2010-11-1-r4)
Supplement: Additional file 3 — Number of unique tiles from the Col-0 genome without UHTS coverage in the four accessions for different tiling array signal ratios. [file gb-2010-11-1-r4-S3.pdf]

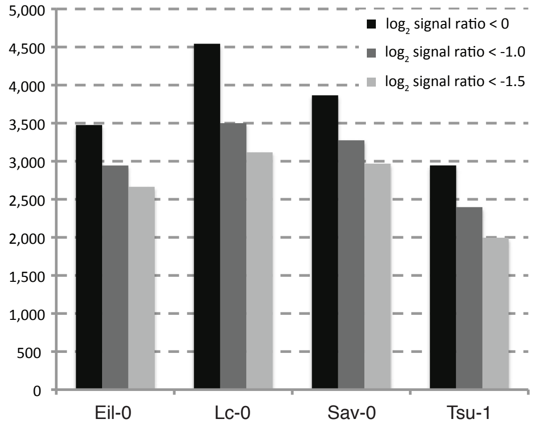

Supplemental Figure 2 Number of unique tiles from the Col-0 genome without UHTS coverage in the four accessions that have a tiling array signal ratio < 0, < -1.0, or < -1.5. Only tiles located within transcript regions were considered.
